# Differences in HCV Viral Decline between Low and Standard-Dose Pegylated-Interferon-Alpha-2a with Ribavirin in HIV/HCV Genotype 3 Patients

**DOI:** 10.1371/journal.pone.0048959

**Published:** 2012-11-08

**Authors:** Antonio Rivero-Juárez, Luis F. Lopez-Cortes, Angela Camacho, Almudena Torres-Cornejo, Juan A. Pineda, Manuel Marquez-Solero, Antonio Caruz, Rosa Ruiz-Valderas, Julian Torre-Cisneros, Alicia Gutierrez-Valencia, Antonio Rivero

**Affiliations:** 1 Unit of Infectious Diseases, Hospital Universitario Reina Sofia, Cordoba, Spain; 2 Maimonides Institute for Biomedical Research, Cordoba, Spain; 3 Enfermedades Infecciosas, Microbiología y Medicina Preventiva, Instituto de Biomedicina de Sevilla, Hospital Universitario Virgen del Rocío/Consejo Superior de Investigaciones Científicas/Universidad de Sevilla, Seville, Spain; 4 Unit of Infectious Diseases, Hospital Universitario de Valme, Seville, Spain; 5 Unit of Infectious Diseases, Hospital Universitario Virgen de la Victoria, Malaga, Spain; 6 Immunogenetics Unit, Faculty of Sciences, Universidad de Jaén, Jaen, Spain; Temple University School of Medicine, United States of America

## Abstract

**Background:**

The aim of the study was to analyze the different impact of standard and low-dose Peg-IFN-α2a/RBV therapies on HCV viral decline in HIV/HCV genotype 3 co-infected patients during the first weeks of treatment.

**Methods:**

Plasma HCV viral decline was analyzed between baseline and weeks 1, 2 and 4 in two groups of treatment-naïve HCV genotype 3 patients with HIV co-infection. The Standard Dose Group (SDG) included patients who received Peg-IFN at 180 µg/per week with a weight-adjusted dose of ribavirin; Low-Dose Group (LDG) patients received Peg-IFN at 135 µg/per week with 800 mg/day ribavirin. The effect of IL28B genotype on HCV viral decline was evaluated in both groups. HCV viral decline was analyzed using a multivariate linear regression model.

**Results:**

One hundred and six patients were included: 48 patients in the SDG and 58 in the LDG. HCV viral decline for patients in the LDG was less than for those in the SDG (week 1∶1.72±0.74 log_10_ IU/mL *versus* 1.78±0.67 log_10_ IU/mL, p = 0.827; week 2∶2.3±0.89 log_10_ IU/mL *versus* 3.01±1.02 log_10_ IU/mL, p = 0.013; week 4∶3.52±1.2 log_10_ IU/mL *versus* 4.09±1.1 log_10_ IU/mL, p = 0.005). The linear regression model identified the Peg-IFN/RBV dose as an independent factor for HCV viral decline at week 4.

**Conclusions:**

Our results showed that HCV viral decline was less for patients in the low-dose group compared to those receiving the standard dose. Until a randomized clinical trial is conducted, clinicians should be cautious about using lower doses of Peg-IFN/RBV in HIV/HCV genotype 3 co-infected patients.

## Introduction

The best determinant of response to hepatitis C virus (HCV) treatment using pegylated-interferon (Peg-IFN) in combination with ribavirin (Peg-IFN/RBV) is the HCV genotype itself. Rates of sustained virological response (SVR) in genotypes 1 or 4 HCV patients co-infected with HIV vary between 17 and 46% in different studies, while for genotype 3 HCV/HIV co-infected patients these values range between 43 and 71% [1], [2]. The higher treatment response rate for genotype 3 HCV patients is due the higher rate of HCV viral clearance in the first weeks of therapy [3]. On the other hand, it has also been observed that HCV genotype 3 patients progress more rapidly to advanced stages of liver fibrosis and hepatic steatosis and are at a significantly higher risk of developing hepatocellular carcinoma [4]–[6]. For these reasons, patients infected with HCV genotype 3 constitute a population which requires the early implementation of treatment.

The current recommended treatment for genotype 3 HCV/HIV co-infected patients is 48 weeks with Peg-IFN-α2a or Peg-IFN-α2b (180 µg/kg and 1.5 µg/kg per week, respectively) combined with ribavirin (weight-adjusted) [7], [8]. However, neither duration of treatment nor drug dose have been clearly optimized. For genotype 3 HCV monoinfected patients, the recommended length of treatment is 24 weeks, and even 12–16 weeks for patients achieving rapid virological response (RVR), and lower doses of Peg-IFN/RBV have been shown to achieve similar SVR rates to the standard dose [9]–[11]. However, because of the different responses to treatment, more rapid progression to liver fibrosis, interaction with antiretroviral treatment drugs and immunological characteristics, the results cannot be extrapolated to HIV/HCV co-infected patients [12], [13].

Early viral kinetics gives clinicians valuable information about the outcome of HCV treatment. Several studies have shown that HCV viral decay in the first weeks after start of treatment can help identify which patients will respond to treatment and which will not [14]–[19]. HCV viral decline during the first weeks of treatment is also useful for analyzing the effects of different drug doses, including the impact on HCV clearance.

The aim of this study was to analyze the different impacts of standard and lower-than- standard dose Peg-IFN-α2a/RBV therapy on HCV viral decline in HIV/HCV genotype 3 co-infected patients during the first weeks after start of treatment.

## Methods

### Patients, Study Design and Treatment Regimen

Two groups of HIV/HCV genotype 3 co-infected patients who were naïve to HCV treatment were included in the study.

The Standard Dose Group (SDG) included patients enrolled in a prospective study designed to evaluate the efficacy of a treatment strategy for chronic hepatitis C genotype 3, who were administered Peg-IFN-α2a at 180 µg/per week combined with a weight-adjusted dose of ribavirin (1000 mg/day for <75 kg, 1200 mg/day for ≥75 kg); length of treatment (24 or 48 weeks) was determined according to whether or not RVR was achieved.

The Low Dose Group (LDG) included patients enrolled in an open-label, single-arm clinical trial (Reference: NCT00553930) evaluating the efficacy of Peg-IFN-α2a at a dose of 135 µg/per week combined with a daily ribavirin dose of 800 mg.

### Ethical Aspects

The SDG study was designed and carried out according to the Helsinki declaration and was approved by the Ethics Committee of the Hospital Reina Sofia of Cordoba. (Reference: NCT00553930). All patients were informed and signed an informed consent form before participating in the study. The protocol of the LDG trial and supporting CONSORT checklist are available as supporting information; see [20]. The study protocol was approved by the Agencia Española del Medicamento and a central ethics committee (Comité Autonómico de Ensayos Clínicos, Consejería de Salud, Junta de Andalucía). The study was conducted according to the Declaration of Helsinki and current guidelines on Good Clinical Practices. This trial is registered at NIH register (ClinicalTrials.gov: NCT00553930) and EMEA (N°EudraCT: 2007-000814-35).

### Data Collection

Host, clinical and virological characteristics were collected. Fibrosis stage was determined by biopsy or liver transient elastography (FibroScan®, Echosen. Paris). Significant fibrosis was defined as a METAVIR fibrosis score of F2–F4 in liver biopsy or a liver stiffness value of ≥8.9 kPa [21].

### Virological Evaluation

Plasma HCV RNA load measurements were conducted at baseline and weeks 1, 2 and 4, using a quantitative PCR assay (Cobas TaqMan, Roche Diagnostic Systems Inc., Pleasanton, CA, USA) and using a detection limit of 15 IU/mL. Viral load was expressed as log_10_IU/mL.

### IL28B Genotyping

DNA was extracted using the automated MagNA Pure DNA extraction method (Roche Diagnostics Corporation. Indianapolis, IN 46250, USA). Single nucleotide polymorphism (SNP) rs129679860, located 3 kilobases upstream of the IL28B, and in strong linkage disequilibrium with a non-synonymous coding variant in the IL28B gene (213A>G, K70R; rs81031142), was genotyped. Genotyping was carried out using a custom TAQMAN assay (Applied Biosystems, Foster City, California, USA) on DNA isolated from whole blood samples, on a Stratagene MX3005 thermocycler with MXpro software (Stratagene, La Jolla, California, USA), following the manufacturer’s instructions. The researchers responsible for genotyping were blinded to other patient data. The IL28B genotype was defined as CC or non-CC (TT/CT).

### Statistical Analysis

Continuous variables were expressed as mean ± standard deviation or median (Q1–Q3) and were analyzed by the Student’s *t* test, Mann-Whitney *U*-test or Kruskal-Wallis test. Categorical variables were expressed as numbers of cases (percentage). Frequencies were compared using the χ^2^ test or Fisher’s exact test. Significance was defined as a *p* value of less than 0.05. Plasma HCV RNA decline, according to SDG and LDG dose, was analyzed from baseline to weeks 1, 2 and 4. The effect of IL28B genotype on HCV viral decline was also analyzed in both treatment groups. Patients presenting an undetectable HCV viral load at any time point during the study were excluded when calculating reduction of HCV RNA levels at a later time. A multivariate linear regression model was used to analyze HCV viral decline between baseline and the various time points. In addition, two linear regression models of HCV viral decline from baseline to the different time points were analyzed according to treatment group. The analysis was performed using the SPSS statistical software package, version 18.0 (IBM Corporation, Somers, NY, USA).

## Results

One hundred and six HIV/HCV genotype 3 co-infected patients were included in the study. Forty-eight (45.3%) patients were included in the SDG and 58 (54.7%) in the LDG. The baseline population characteristics of the two groups are shown in Table 1 .

**Table 1:** Baseline Population Characteristics.

Characteristic	SDG	LDG	P
N	48	58	
Male. N (%)	39 (81.2)	51 (87.9)	0.379
Age (years). Mean (SD)	40.4 (8.86)	43.6 (5.37)	0.612
Use of HAART, n (%)	45 (93.7)	52 (89.6)	0.588
AIDS diagnosis in the past, n (%)	13 (27.1)	10 (17.2)	0.213
Baseline CD4 count (cells/mm^3^). mean (SD)	479.9 (234.7)	496.5 (266.4)	0.743
PIDU, n (%)	43 (89.5)	48 (82.7)	0.454
HCV baseline viral load (log_10_ IU/mL). Mean (SD)	5.72 (0.75)	5.51 (1)	0.247
Liver fibrosis stage F2–F4. n (%)	32 (66.6)	33 (56.9)	0.277
Liver Cirrhosis. n (%)	12 (25)	21 (36.2)	0.253
ALT (IU/L). Mean (SD)	80.3 (47.7)	91 (57)	0.409
AST (IU/L). Mean (SD)	65.5 (35.2)	79.9 (33)	0.541
Total fasting cholesterol (mg/dL). Mean (SD)	156.1 (40.4)	147 (37)	0.325
LDL cholesterol (mg/dL). Mean (SD)	86.8 (33.6)	76 (29)	0.147
Platelet count (10^3^/µL). Mean (SD)	183 (65)	176 (68)	0.654
IL28B-CC genotype. N (%)	13 (43.4)^†^	24 (53.3)^‡^	0.409

Standard drug dose group (SDG); low drug dose group (LDG); human immunodeficiency virus (HIV); highly active antiretroviral treatment (HAART); acquired immunodeficiency syndrome criteria in the past (AIDS); previous intravenous drug user (PIDU); hepatitis C virus (HCV); interleukin 28B (IL28B). ^†^Available for 30 patients. ^‡^Available for 45 patients.

### HCV Viral Decline According to Treatment Group

**Figure 1:**
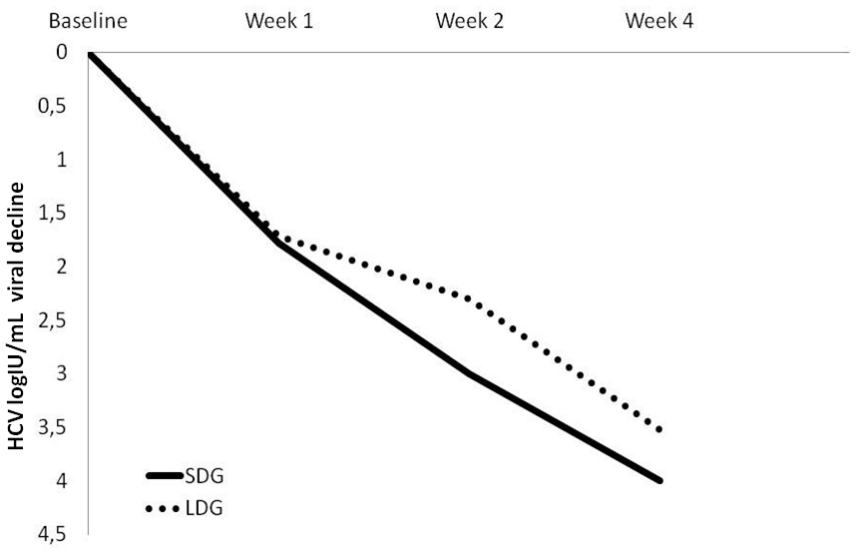
Mean HCV viral decline between baseline and weeks 1, 2 and 4 for the standard drug dose group (SDG) and the low drug dose group (LDG).

HCV viral decline of patients given the lower dose treatment was less than for those in the SDG, at weeks 2 and 4 after start of treatment, although not at week 1 (week 1∶1.72±0.74 log_10_ IU/mL *versus* 1.78±0.67 log_10_ IU/mL, p = 0.827; week 2∶2.3±0.89 log_10_ IU/mL *versus* 3.01±1.02 log_10_ IU/mL, p = 0.013; week 4∶3.52±1.2 log_10_ IU/mL *versus* 4.09±1.1 log_10_ IU/mL, p = 0.005) (Figure 1). The multivariate linear regression models of factors associated with HCV viral decline at weeks 1, 2 and 4 showed that the steeper and sustained HCV viral decline from week 2 to week 4 was associated with a lower baseline HCV RNA viral load and with patients in the SDG (Table 2). IL28B-CC and HCV viral decline were not associated.

**Table 2:** Multivariate linear regression model of HCV viral decline between baseline and weeks 1, 2 and 4 after start of treatment.

HCV decline at week 1				
Factor	Condition	B		P
Treatment Group	SDG	−0.175		0.327
Baseline HCV viral load (log_10_ IU/mL)		0.059		0.637
Significant liver fibrosis stage	Yes	−0.247		0.676
IL28B genotype	Non-CC	0.296		0.113
**HCV decline at week 2**				
**Factor**	**Condition**	**B**	**P**	
Treatment Group	SDG	0.274	**0.037**	
Baseline HCV viral load		0.329	**0.026**	
Significant liver fibrosis stage	Yes	−0.113	0.340	
IL28B genotype	Non-CC	−0.103	0.551	
**HCV decline at week 4**				
**Factor**	**Condition**	**B**	**P**	
Treatment Group	SDG	0.335	**0.025**	
Baseline HCV viral load		0.339	**0.002**	
Significant liver fibrosis stage	Yes	−0.180	0.092	
IL28B genotype	Non-CC	−0.041	0.791	

adjusted coefficient (B), hepatitis C virus (HCV), interleukin 28B (IL28B), standard drug dose group (SDG). R^2^ = 0.327.

### HCV Viral Decline According to Treatment Group and IL28B Genotype

**Figure 2:**
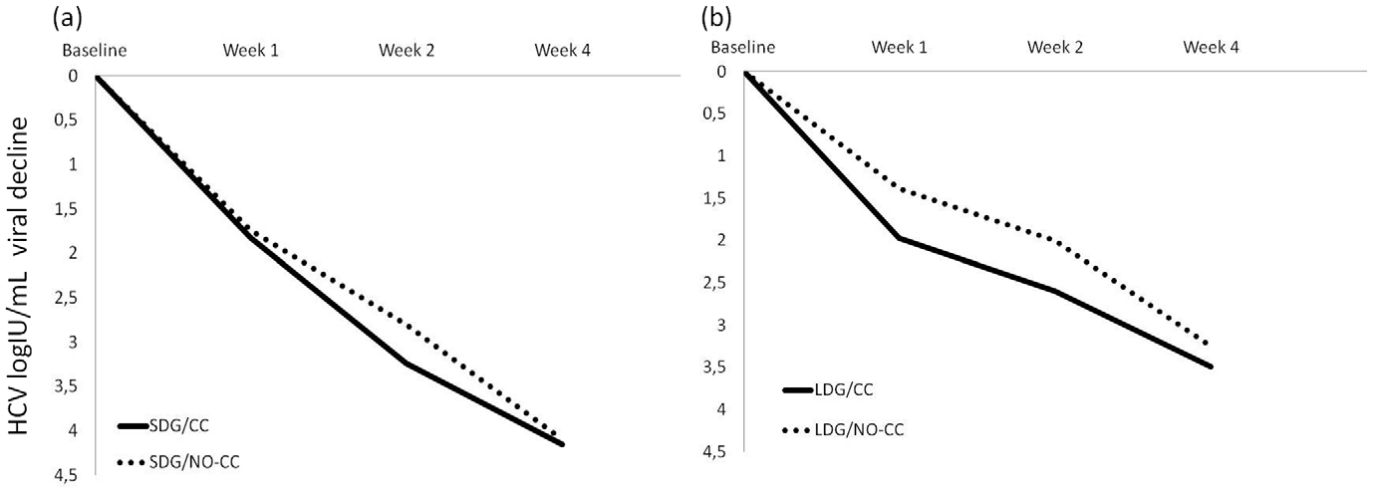
Mean HCV viral decline by IL28B genotype for the standard dose group (SDG) (**Figure 2A**) and the low-dose group (LDG) (**Figure 2B**).

Among SDG patients, there were no differences of HCV viral decline by IL28B genotype at week 1 (1.82±0.91 log_10_ IU/mL *versus* 1.74±0.87 log_10_ IU/mL, p = 0.852), week 2 (3±1.1 log_10_ IU/mL *versus* 3.08±1.09 log_10_ IU/mL, p = 0.807) or week 4 (4.14±0.84 log_10_ IU/mL *versus* 4.17±1.06 log_10_ IU/mL, p = 0.938) (Figure 2A). Similarly, no differences of HCV viral decline by IL28B genotype were found among LDG patients at week 1 (2.07±0.47 log_10_ IU/mL *versus* 1.42±1.08 log_10_ IU/mL, p = 0.071), week 2 (2.46±0.86 log_10_ IU/mL *versus* 2.03±1.35 log_10_ IU/mL, p = 0.257) or week 4 (3.44±0.94 log_10_ IU/mL *versus* 3.26±1.01 log_10_ IU/mL, p = 0.573) (Figure 2B).

Among IL28B-CC genotype patients, HCV viral decline was greater in the SDG than in the LDG at weeks 2 and 4, but not at week 1 (week 1: p = 0.362; week 2: p = 0.051; week 4: p = 0.033). Likewise, HCV viral decline was greater among SDG patients carrying the IL28B non-CC genotype than among their LDG non-CC counterparts, at weeks 2 and 4 (week 1: p = 0.343; week 2: p = 0.034; week 4: p = 0.037).

### Rapid Virological Response Rate

HCV viral load for 6 (5.6%) patients could not be evaluated at week 4. Of the remaining 100 patients, 66 (66%) achieved RVR: thirty-five (72.9%) in the SDG, and 31 (59.6%) in the LDG (p = 0.174). RVR rates by treatment group and IL28B genotype are shown in Table 3.

**Table 3:** Rapid virological response (RVR) rate by treatment group and IL28B genotype.

Treatment group	IL28B genotype	RVR. N (%)	P
SDG	CC	9 (40.9)	0.748
	Non-CC	12 (44.4)	
LDG	CC	13 (38.2)	0.642
	Non-CC	12 (35.3)	
**IL28B genotype**	**Treatment group**	**RVR. N (%)**	**P**
CC	SDG	9 (40.9)	0.642
	LDG	13 (38.2)	
Non-CC	SDG	12 (44.4)	0.221
	LDG	12 (35.3)	

Interleukin 28B (IL28B), rapid virological response (RVR), standard drug dose group (SDG), low drug dose group (LDG).

## Discussion

In our study, HCV viral decay of patients who received low-dose Peg-IFN/RBV treatment was less during the first weeks of treatment than for those receiving the standard Peg-IFN/RBV dose. This finding suggests that a lower Peg-IFN/RBV dose has less antiviral activity than the standard dose.

High viral decline during the first weeks of treatment leads to a high RVR rate [3]. Several factors have been identified as determining HCV viral decay [3], [21]. Our study found that HCV viral decay correlated with Peg-IFN dose in HIV/HCV genotype 3 co-infected patients, with steeper viral decline from week 2 to week 4 in the SDG compared to the LDG. Our study also found that the dose of Peg-IFN administered during treatment was the most important factor affecting HCV viral decline in these patients. RVR rates in our study were also higher in the SDG compared to the LDG (72.9% *versus* 59.6%), although the differences between the two groups were not statistically significant. This point should be interpreted with caution, since a better powered cohort might be required for statistically significant associations, due to the high RVR rate among HCV genotype 3 patients.

Reducing the dose in drug-based HCV therapy for monoinfected HCV genotype 3 patients has been studied in various clinical trials. Firstly, reducing the dose of Peg-IFNα2a from 180 µg/per week to 135 µg/per week was shown to give similar RVR and SVR rates [9], [10]. Secondly, SVR rates did not differ significantly according to whether a lower daily dose or a weight-adjusted dose of ribavirin was used [22]–[24]. A reduced dose of both drugs is, therefore, applicable to this patient population.

However, in HCV genotype 3 patients co-infected with HIV, a low-dose Peg-IFN/RBV combination would have a considerable impact, in terms of a high SVR, greater cost savings and fewer adverse events than the standard dose. A previous open-label, single-arm pilot clinical trial involving 58 HCV/HIV co-infected patients receiving low doses of Peg-IFN/RBV found that SVR rates were 58.3% based on intention-to-treat [20]. The main limitation of this study was the fact that it was not randomized but a single-arm study whose results were compared with those observed in earlier clinical trials The results of the pilot study suggested that a lower Peg-IFN/RBV dose might be as effective as the standard dose, so supporting the design of a randomized controlled trial. There are no data however about the efficacy or safety of standard Peg-IFN/RBV dose compared to a lower-than standard dose.

To the best of our knowledge, this is the first study to compare the efficacy of HCV viral clearance using low-dose and standard-dose drug therapy in HIV/HCV co-infected patients in the first weeks after treatment starts. Our findings suggest that the antiviral activity of the lower Peg-IFN/RBV dose is weaker than with the standard dose, which does not support equating the two for HIV/HCV co-infected patients. Our results also suggest that HCV viral decline during the first weeks of treatment would be dose-dependent, although the mechanism responsible for the difference is unknown.

In our study, we found no relation between IL28B genotype and viral decline in either of the regimens evaluated. The positive effect on treatment response associated with the IL28B-CC genotype has only been observed in patients bearing genotype 1/4 [25], [26]. In fact, a previous study developed by our group reported that variations in IL28B do not have a positive impact on HCV viral decline in the first weeks after start of therapy using a standard drug dose [3]. Nor did IL28B-CC have a positive effect on RVR or SVR at standard or low drug doses in patients bearing genotype 3 [20], [25].

Our study has several limitations. Firstly, our study is not a randomized clinical trial, and the presence of significant bias cannot therefore be ruled out. Secondly, due to the higher RVR rate in HCV genotype 2/3 patients, our study did not have the statistical power to detect differences in RVR rate by drug treatment dose. Thirdly, our study looked at the impact of IL28B by determining only SNP rs12979860, although the impact observed for this is not expected to be different from the other known IL28B SNP (rs8099917).

In conclusion, our results show that patients who received 135 µg/per week with a 800 mg/day ribavirin dose had less HCV viral decline in the first weeks after treatment started than those who received 180 µg/per week with a weight-adjusted ribavirin dose. The implications of weaker HCV viral decline in terms of treatment outcome are unknown, although it would be expected for a weaker HCV decline to lead to a lower RVR and consequently to a lower SVR. In order to resolve this point, our findings provide justification for the design of a randomized clinical trial to compare the specific efficacy endpoints of the two Peg-IFN/RBV doses in HIV/HCV co-infected patients. Until such a randomized clinical trial with these specific endpoints is conducted, therefore, clinicians should be cautious about using lower-than-standard Peg-IFN/RBV doses in HIV/HCV genotype 3 co-infected patients.
